# Transient Laterality of Cerebral Oxygenation Changes in Response to Head-of-Bed Manipulation in Acute Ischemic Stroke

**DOI:** 10.3390/jcm8101739

**Published:** 2019-10-19

**Authors:** Naoki Katayama, Keiichi Odagiri, Akio Hakamata, Naoki Inui, Katsuya Yamauchi, Hiroshi Watanabe

**Affiliations:** 1 Department of Clinical Pharmacology and Therapeutics, Hamamatsu University School of Medicine, 1-20-1 Handayama, Higashi-ku, Hamamatsu 431-3192, Japan; katayama_20@yahoo.co.jp (N.K.); hakamata@hama-med.ac.jp (A.H.); inui@hama-med.ac.jp (N.I.); hwat@hama-med.ac.jp (H.W.); 2 Department of Rehabilitation Medicine, Seirei Mikatahara General Hospital, 3453 Mikatahara-cho, Kita-ku, Hamamatsu 433-8558, Japan; 3 Department of Rehabilitation Medicine, Hamamatsu University Hospital, 1-20-1 Handayama, Higashi-ku, Hamamatsu 431-3192, Japan; yamakatu@hama-med.ac.jp

**Keywords:** cerebral blood volume, hemodynamics, near-infrared spectroscopy, optical imaging, rehabilitation, stroke

## Abstract

Background: Cerebral oxygenation monitoring provides important information for optimizing individualized management in patients with acute ischemic stroke (AIS). Although changes in cerebral oxygenation are known to occur in response to head-of-bed (HOB) elevation within 72 h after onset, changes in cerebral oxygenation during stroke recovery are unclear. We compared changes in total- (tHb), oxygenated- (HbO_2_), and deoxygenated-hemoglobin (deoxyHb) concentrations in response to HOB manipulation between the timeframes within 72 h and 7–10 days after AIS onset. Methods: We measured forehead ΔtHb, ΔHbO_2_, and ΔdeoxyHb in response to HOB elevation (30°) within 72 h (first measurement) and 7–10 days (second measurement) after AIS onset using time-resolved near-infrared spectroscopy. Results: We enrolled 30 participants (mean age 72.8 ± 11.3 years; 13 women) with a first AIS. There were no significant differences in ΔtHb, ΔHbO_2_, or ΔdeoxyHb measurements on the infarct or contra-infarct side. At the first measurement, ΔtHb, ΔHbO_2_, and ΔdeoxyHb measured on the contra-infarct side did not correlate with those measured on the infarct side: ΔtHb (*r* = 0.114, *p* = 0.539); ΔHbO_2_ (*r* = 0.143, *p* = 0.440); ΔdeoxyHb (*r* = 0.227, *p* = 0.221). Notably, at the second measurement, correlation coefficients of ΔtHb and ΔHbO_2_ between the contra-infarct and infarct sides were statistically significant: ΔtHb (*r* = 0.491, *p* = 0.008); ΔHbO_2_ (*r* = 0.479, *p* = 0.010); ΔdeoxyHb (*r* = 0.358, *p* = 0.054). Conclusion: Although changes in cerebral oxygenation in response to HOB elevation had a laterality difference between hemispheres within 72 h of AIS onset, the difference had decreased, at least partially, 7–10 days after AIS onset.

## 1. Introduction

Acute ischemic stroke (AIS) is a significant cause of permanent disability [1]. Early rehabilitation for AIS patients—considered an important issue in poststroke functional outcomes—has been recommended in recent guidelines [2,3]. A large-scale clinical trial; however, provided evidence that early intervention was not associated with disability outcomes [4]. It is also recognized that supine AIS patients have improved cerebral blood flow (CBF) and oxygenation, although with an increased risk of aspiration pneumonia [5,6,7,8]. Another clinical trial revealed that a head-up position initiated within 24 h of AIS onset was not associated with a disability outcome or severe adverse effects, including pneumonia [9]. Thus, the optimal head position in patients with AIS is still unknown. 

Near-infrared spectroscopy (NIRS) noninvasively measures hemoglobin (Hb) levels in the brain [10]. Compared with other technologies, such as transcranial Doppler (TCD) and positron emission tomography (PET), NIRS has several advantages: (1) it allows flexible measurements in sitting, standing, and moving subjects; (2) it is an irradiation-based, completely noninvasive technique that does not cause adverse effects on the body during repeated measurements, even in children; (3) it has high time resolution; and (4) it is compact and portable. Because of these advantages, the use of NIRS, such as cerebral oxygen monitors, is increasing in the medical field despite its drawbacks (e.g., possible interferences caused by attachment of optodes, shallow measurement depth, effects of drugs influencing cerebral blood flow or cutaneous blood flow, artifacts of cutaneous blood flow, and narrow measurement territory depending on the attachment site of optode). This increase is because these systems are simple but enable the observation of changes in brain activity over time via monitoring of Hb levels, which reflect fluctuations in regional cerebral blood flow. Indeed, NIRS can be useful to detect the intraindividual fluctuation and the interindividual difference of cerebral hemodynamic response in response to posture change [11]. Thus, well-known applications of NIRS include the monitoring of cerebral blood flow and hypoxic conditions in a variety of clinical settings [12,13,14]. During the last two decades, several studies have used the NIRS system to evaluate changes in cerebral oxygenation in upright AIS patients [7,15,16,17]. Their findings provided important information for optimal individualized management, based on cerebral oxygenation monitoring in AIS patients. Nevertheless, correlation of the total- (tHb), oxygenated- (HbO_2_), and deoxygenated-hemoglobin (deoxyHb) concentrations in response to head-of-bed (HOB) elevation between the infarct and contra-infarct sides have never been assessed, leaving the changes in cerebral oxygenation during stroke recovery not well understood. In the current exploratory study, we therefore aim to compare the changes in tHb (ΔtHb), HbO_2_ (ΔHbO_2_), and deoxyHb (ΔdeoxyHb) in response to HOB manipulation between the timeframes within 72 h and 7–10 days after AIS onset.

## 2. Methods

### 2.1. Study Design and Participants

This study was designed as a single-center exploratory study to compare the changes in cerebral oxygenation in response to HOB manipulation between different time points in AIS patients, and it was conducted at Seirei Mikatahara General Hospital, Hamamatsu, Japan. Study participants were consecutively recruited from among acute cerebral infarction patients hospitalized at our hospital from September 2016 to March 2017. Eligible patients were those having a first-ever ischemic stroke and who had been hospitalized within 24 h of symptoms onset. Main exclusion criteria were a patient with infratentorial stroke; a history of cerebral disease (prior stroke, brain contusion, brain tumor, brain infections, intracerebral hemorrhage and trauma); orthostatic hypotension; taking antihypertensive agents after hospitalization; or unable to participate in this study (could not maintain a 30° passive sitting position; presence of a skin disease (not suitable for applying a probe to the forehead); unable to follow verbal instructions).

### 2.2. Ethics and Study Registration

This study protocol complied with the Helsinki Declaration. The institutional research review board of Seirei Mikatahara General Hospital and Hamamatsu University School of Medicine approved the study (Approved number 16-15 and 16-001). Written informed consent was provided by all participants. The study was registered at the UMIN Clinical Trials Registry (URL: http://www.umin.ac.jp/ctr/index.htm. Unique identifier: UMIN 000022904).

### 2.3. Cerebral Hemoglobin Concentration Measurement by Time-Resolved NIRS

We used a single-channel, time-resolved NIRS system (TRS-10; Hamamatsu Photonics K.K., Hamamatsu, Japan) to measure bilateral forehead cerebral (prefrontal cortex) hemoglobin concentration. The temporal profile obtained from TRS-10 measurement was fitted with that obtained from the theoretical solution of the photon diffusion equation (DE) [18], because the DE-fit method could provide information about the hemodynamic changes in the depth direction [19]. The TRS-10 system consists of three pulsed laser diodes with wavelengths of 759, 797, and 833 nm, having a duration of 100 ps and repetition frequency of 5 MHz. An optode, which includes infrared light irradiation and reception probes in a single device, was fixed on the participant’s head with Velcro and a headband so the irradiated infrared light was positioned at Fp1 and Fp2 according to the International 10-20 system. This NIRS device can measure the tHb, HbO_2_, and deoxyHb of tissues within a semicircular area between the irradiation and reception probes. The measurement depth increases with increased distance between the irradiation and reception probes (limit of 5 cm), because the farther the distance, the weaker the light reaching the reception probe. One study reporting simultaneous measurements with TRS-10 and PET found that TRS-10 measurements with irradiation and reception probes 3 cm apart significantly correlated with PET measurements around gray matter [20]. We therefore set the distance between the irradiation and reception probes at 3 cm outside the infrared reception port on the optode.

### 2.4. Cerebral Blood Hemoglobin Concentration Measurements Protocol

Based on previous studies [21,22,23,24], we measured forehead tHb, HbO_2_, and deoxyHb within 72 h (first measurement) and 7–10 days (second measurement) after AIS onset. After placing probes on the forehead, the participant laid on his/her back. Data were collected every 10 s for 5 min at each HOB angle (0°, 30°, 0°) sequentially. At each HOB angle, the mean tHb, HbO_2_, and deoxyHb values were calculated after discarding data obtained during the first minute, because it took 15 s to change the HOB position of the bed. Because the TRS-10 system has a single channel, two consecutive measurements were conducted in each participant. We first measured forehead tHb, HbO_2_, and deoxyHb on the contra-infarct side and then on the infarct side. Systemic blood pressure and heart rate were also measured for 1 min in each position using an automatic hemodynamometer (HBP1300; Omron Corp., Tokyo, Japan).

### 2.5. Statistical Analysis

Values are expressed as means ± standard deviations (SD) or medians (interquartile range) (nonparametrically distributed values) of the indicated numbers or proportions (%). Changes in systemic blood pressure, heart rate, tHb, HbO_2_, and deoxyHb were compared with the baseline (HOB 0°). These measurement values at HOB 30° were compared with those at HOB 0° using the Wilcoxon signed-rank test. Correlations between the infarct and contra-infarct sides for each measurement were assessed using Spearman’s rank correlation coefficient. The significance of the difference between the two correlation coefficients was evaluated using the Fisher r-to-z transformation. *p* < 0.05 was regarded as indicating statistical significance. All statistical analyses were performed using PASW Statistics version 18.0.0 (IBM Co., Armonk, NY, USA) and Microsoft Excel 2016 (Microsoft Co., Redmond, WA, USA).

## 3. Results

### 3.1. Study Participants’ Characteristics

Altogether, 32 AIS patients met the inclusion criteria and were enrolled. Two participants died before the second measurements and were excluded from the analyses. The participants’ characteristics are shown in Table 1.

### 3.2. Changes in Blood Pressure, Heart Rate, tHb, HbO_2_, and deoxyHb with HOB Elevation

Table 2 shows the changes in systolic (SBP) and diastolic (DBP) blood pressures and the heart rate in response to HOB elevations from 0° to 30°. These HOB elevations did not affect any hemodynamic parameters. There were also no intraindividual differences in the SBP or heart rate at baseline measurements (HOB 0°) at each measurement session (first measurement: SBP (*p* = 0.74), DBP (*p* = 0.87), heart rate (*p* = 0.94); second measurement: SBP (*p* = 0.83), DBP (*p* = 0.94), heart rate (*p* = 0.30).

### 3.3. Changes in Cerebral Hemoglobin Concentrations with HOB Elevation

**Figure 1:**
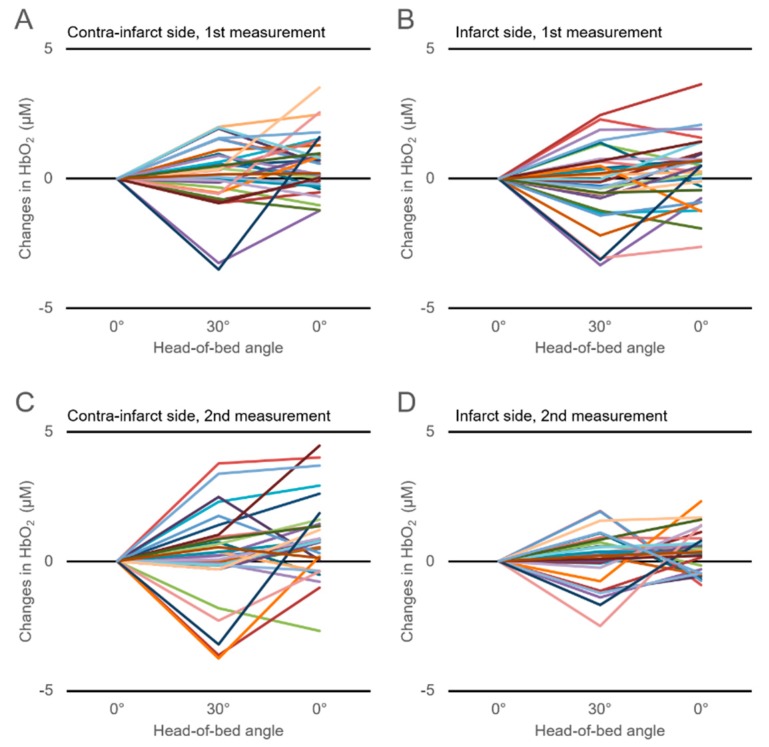
Time-series changes in the cerebral total hemoglobin (tHb) concentration in response to head-of-bed manipulation (from 0° to 30°) for 30 participants within 72 h of onset of acute ischemic stroke (AIS) (first measurement) on the contra-infarct (contralateral) side (**A**) and infarct side (**B**) and those measured 7–10 days after onset of AIS (second measurement) on the contra-infarct side (**C**) and infarct side (**D**).

**Figure 2:**
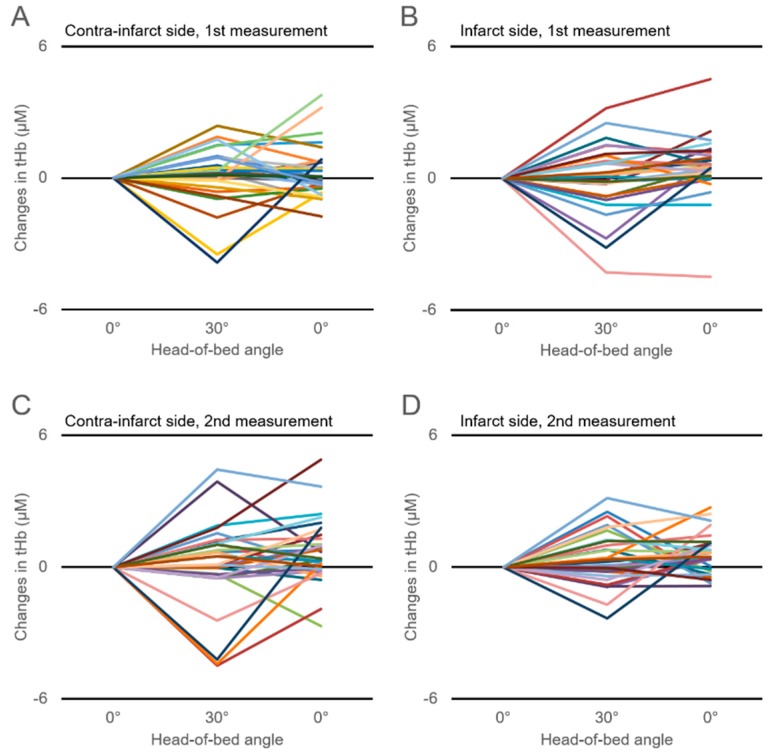
Time-series changes in the cerebral oxygenated-hemoglobin (HbO_2_) concentration in response to head-of-bed manipulation for 30 participants within 72 h of AIS onset (first measurement) on the contra-infarct side (**A**) and infarct side (**B**) and those measured 7–10 days after AIS onset (second measurement) on the contra-infarct side (**C**) and infarct side (**D**).

**Figure 3:**
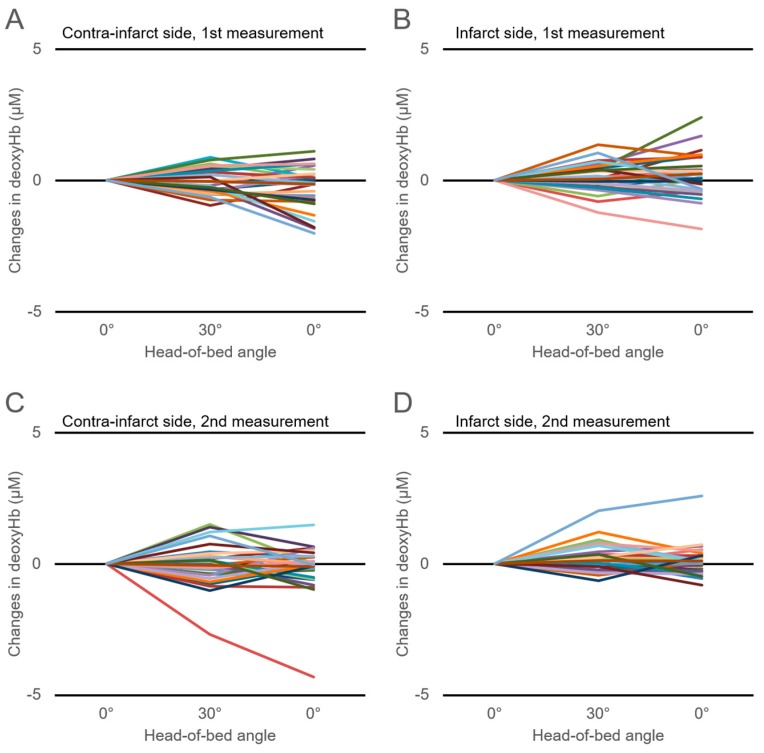
Time-series changes in the cerebral deoxygenated-hemoglobin (deoxyHb) concentration in response to head-of-bed manipulation for 30 participants within 72 h of AIS onset (first measurement) on the contra-infarct side (**A**) and infarct side (**B**) and those measured 7–10 days after onset of AIS (second measurement) on the contra-infarct side (**C**) and infarct side (**D**).

Figure 1 shows the time-series changes in the tHb in response to HOB manipulation. Changes in the tHb showed large interindividual differences, which were also observed in the changes in the HbO_2_ and deoxyHb (Figure 2; Figure 3). There were no significant differences in the ΔtHb, ΔHbO_2_, or ΔdeoxyHb on either the infarct or the contra-infarct side between the two measurements.

### 3.4. Correlations of Hemoglobin Concentration Changes with HOB Elevation between Measurements

**Figure 4:**
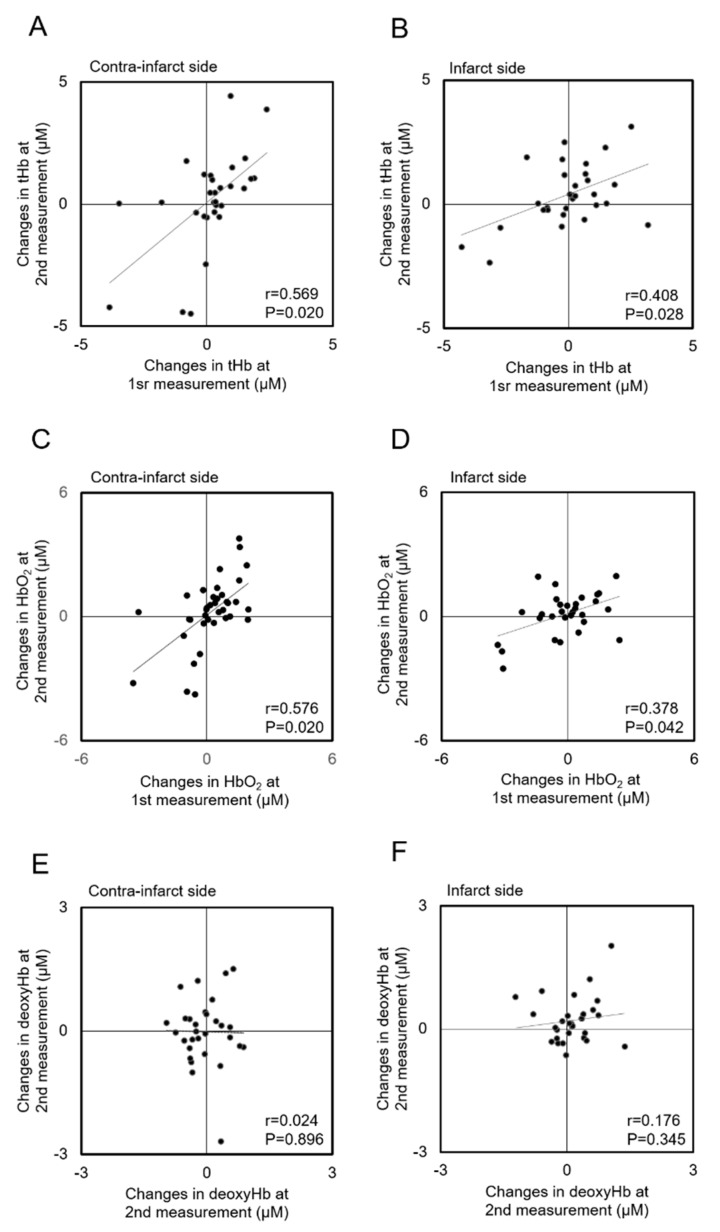
Scatterplots of the changes in total-hemoglobin (tHb) (**A** and **B**), oxygenated-hemoglobin (HbO_2_) (**C** and **D**), and deoxygenated-hemoglobin (deoxyHb) (**E** and **F**) concentrations in response to head-of-bed elevation (from 0° to 30°) between the first and second measurements for each hemisphere.

Figure 4 shows the correlations of the ΔtHb, ΔHbO_2_, and ΔdeoxyHb in response to HOB elevation between the first and second measurements for each hemisphere. The first measurements of the ΔtHb and ΔHbO_2_ were significantly correlated with those at the second measurements for either hemisphere (ΔtHb: contra-infarct side (*r* = 0.569, *p* = 0.020), infarct side (*r* = 0.408, *p* = 0.028); ΔHbO_2_: contra-infarct side (*r* = 0.576, *p* = 0.020), infarct side (*r* = 0.378, *p* = 0.042)). Although correlation coefficients in the contra-infarct side seemed to indicate a stronger relation than those in the infarct side, there were no statistically significant differences for ΔtHb or ΔHbO_2_ (ΔtHb (z = 0.772, *p* = 0.441); ΔHbO_2_ (z = 0.955, *p* = 0.342)), ΔdeoxyHb did not show a significant correlation between the first and second measurements (contra-infarct side (z = 0.024, *p* = 0.896); infarct side (z = 0.176, *p* = 0.345)).

### 3.5. Correlations of Hemoglobin Concentration Changes with HOB Elevation between Infarct and Contra-Infarct Sides

**Figure 5:**
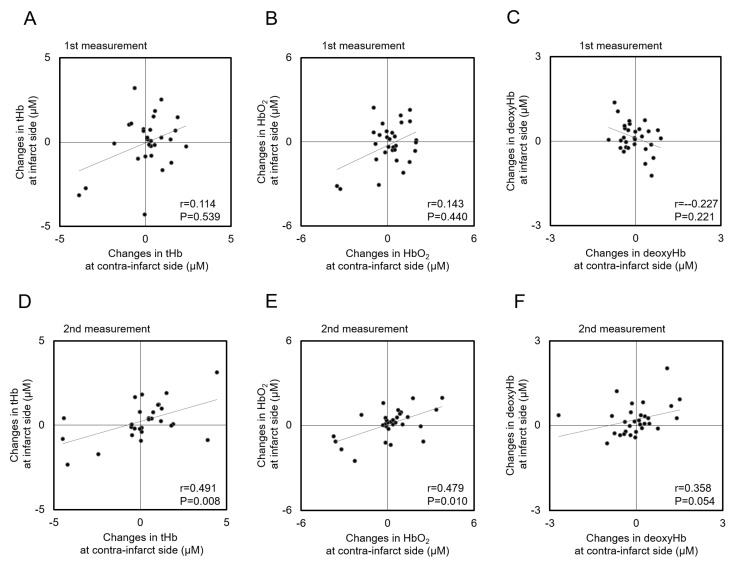
Scatterplots of the changes in total-hemoglobin (tHB), oxygenated-hemoglobin (HbO_2_), and deoxygenated-hemoglobin (deoxyHb) concentrations in response to head-of-bed elevation (from 0° to 30°) at the first (**A**–**C**) and second (**D**–**F**) measurements for each hemisphere.

Figure 5 shows the correlations of ΔtHb, ΔHbO_2_, and ΔdeoxyHb in response to HOB elevation between the infarct and contra-infarct sides. At the first measurement, ΔtHb, ΔHbO_2_, and ΔdeoxyHb measured on the contra-infarct side did not correlate with those on the infarct side (ΔtHb (*r* = 0.114, *p* = 0.539), ΔHbO_2_ (*r* = 0.143, *p* = 0.440); ΔdeoxyHb (*r* = 0.227, *p* = 0.221)) (Figure 5A–C). Notably, the correlation coefficients of ΔtHb, ΔHbO_2_, and ΔdeoxyHb values between the infarct and contra-infarct sides at the second measurement were statistically significant, except for ΔdeoxyHb (ΔtHb (*r* = 0.491, *p* = 0.008); ΔHbO_2_ (*r* = 0.479, *p* = 0.010); ΔdeoxyHb (*r* = 0.358, *p* = 0.054)) (Figure 5D–F).

## 4. Discussion

We believe that this is the first study to assess the effect of gradual HOB manipulation (from 0° to 30°) of forehead hemoglobin concentration in AIS patients, revealed by two measurements using NIRS. There were three main findings of this investigation. (1) The HOB elevation from 0° to 30° did not affect systemic blood pressure or heart rate. ΔtHb, ΔHbO_2_, and ΔdeoxyHb also did not change in response to HOB elevation, although large interindividual variabilities were observed. (2) ΔtHb and ΔHbO_2_ in response to HOB elevation measured within 72 h of AIS onset showed significant correlations with those measured 7–10 days after AIS onset in both hemispheres. (3) Although ΔtHb, ΔHbO_2_, and ΔdeoxyHb measured on the infarct side did not correlate with those measured on the contra-infarct side within 72 h of AIS onset, the correlation coefficients of these NIRS parameters were significantly correlated between the hemispheres 7–10 days after AIS onset. 

It is known that CBF velocity (CBFV) and the total cerebral Hb concentration are reduced in head-up position of healthy subjects and chronic ischemic stroke patients. Furthermore, decreases in CBVF and cerebral Hb concentration could be affected by a drop in systemic blood pressure [5,25,26,27]. In this study, the average blood pressure and heart rate did not change with HOB elevation from 0° to 30°. Neither were there changes in the cohort-averaged tHb, HbO_2_, and deoxyHb concentrations for either hemisphere over two measurements. However, as shown in Figure 1, Figure 2 and Figure 3, changes in tHb, HbO_2_, and deoxyHb showed large differences among individuals. Approximately half of our study patients showed increased cerebral hemoglobin concentrations (the so-called paradoxical response) in response to HOB elevation for either hemisphere over the two measurements. This paradoxical response phenomenon was in line with previous reports that evaluated the effect of head-position changes on cerebral oxygenation in AIS patients [7,16,17]. Several previous studies reported that the paradoxical response was also seen in brain-injured patients but not healthy subjects, suggesting that it is pathological [11,28,29,30]. Although details of the mechanism of the paradoxical response are still unknown, an increasing intracranial pressure, the hemodynamic consequences of heart failure, and an autonomic disturbance have been proposed as causes [17]. Considerably varying individual cerebral oxygenation responses, including the paradoxical response, could explain why the cohort-averaged ΔtHb, ΔHbO_2_, and ΔdeoxyHb concentrations did not change in response to HOB elevation.

Correlations of ΔtHb, ΔHbO_2_, and ΔdeoxyHb concentrations in response to HOB elevation between the infarct and contra-infarct sides had not been reported prior to this study. Thus, we seem to be the first to show that the ΔtHb and ΔHbO_2_ measurements within 72 h of AIS onset significantly correlated with those 7–10 days after AIS onset for either hemisphere. Notably, although the ΔtHb, ΔHbO_2_, and ΔdeoxyHb, in response to HOB elevation, did not show a significant correlation between hemispheres within 72 h of AIS onset, correlations of the ΔtHb, ΔHbO_2_, and ΔdeoxyHb 7–10 days after onset were statistically significant. Although we could not clarify the mechanism of this alteration, various possible explanations are assumed. Changes in cerebral oxygenation variables in response to HOB manipulation reflected CBF volume change. It could be related with gravitational force acting on passively contacted brain vessels in the ischemic territory [31]. It is also possible that systemic hemodynamics changes affect CBF; however, the HOB elevation from 0° to 30° did not affect systemic blood pressure or heart rate. A previous study, which measured cerebral mean flow velocity by TCD, suggested that the effect of BP change in response to head position change was equivocal [31,32,33]. In the current study, HOB elevations did not affect SBP, DBP, or HR, and no intraindividual differences in the SBP or heart rate at baseline measurements (HOB 0°) was observed at each measurement session. While cardiac output or stroke volume of the left ventricle was not measured, we thought that the effect of systemic hemodynamics changes could be limited. It is well-recognized that the brain edema is one of the lethal complications for AIS patients, and it causes a decrease in cerebral perfusion pressure through an increased intracranial pressure (ICP) [34]. Although we did not include AIS patients who received surgical decompression for severe brain edema (because of unsuitability of applying a probe to the forehead) and two participants who died before second measurement (suspected brain edema) were excluded from the analyses, the possibility that a raised ICP could affect the changes in ΔtHb, ΔHbO_2_, and ΔdeoxyHb on the infarct side in response to HOB elevation could not be denied because we did not monitor the ICP in our participants. Development of collateral circulation also affected the changes in ΔtHb, ΔHbO_2_, and ΔdeoxyHb on the infarct side in response to HOB elevation. Our study participants received magnetic resonance angiography (MRA) at the time of admission and 7–10 days after stroke onset. MRA often cannot provide information about the collateral circulation of AIS patients in clinical settings because of motion artifacts and its spatial resolution, whereas recent development of MRA can detect collateral circulation in research settings [35,36]. Furthermore, our study participants did not receive cerebral angiography because they had no indication of thrombolytic therapy at the time of admission. Thus, detailed information of collateral circulation is not available in the current study. Another possible mechanism is the alteration of cerebral autoregulation in AIS patients. Cerebral autoregulation is an inherent process of blood vessels that maintains CBF at a constant level over a wide range of changes in the systemic blood pressure or intracranial pressure. It has been generally accepted that cerebral autoregulation is impaired in patients with AIS [37,38]. Conventionally, TCD has often been used to measure the CBFV to assess cerebral autoregulation [39], and mean flow velocity index of dynamic autoregulation (Mx index) was established as a standard parameter [40,41]. Recent literature; however, has reported that cerebral oxygenation parameters measured by NIRS (e.g., cerebral oxygen saturation, cerebral oxygenation index) are considered surrogates of CBF [42,43,44,45]. In addition, it was reported that the NIRS-derived tHb signal reflects regional changes in cerebral blood volume (CBV), and HbO_2_ correlates with cerebral capillary oxygen saturation [20,46,47,48]. Steiner, L. A. and colleagues demonstrated that tissue oxygen index of dynamic autoregulation (Tox) measured by NIRS (NIRO 200, Hamamatsu Photonics K.K.) significantly correlated with the Mx index [41]. Although NIRS is useful for cerebral autoregulation assessment, we could not make mention of the relationship between our results and cerebral autoregulation. We could not calculate the Tox index because we did not measure continuous blood pressure, and TRS-10 could not directly measure the tissue oxygen index. The literature suggests that AIS severity could influence the degree of spatiotemporal compromise of cerebral autoregulation [21,24,49]. Tutaj et al. reported that cerebral autoregulation was transiently impaired at an infarct hemisphere 1.3 ± 0.5 days after the onset of a large-vessel AIS, recovered at 9.75 ± 2.2 days—which could be in line with our findings [21]. Thus, we speculated the possibility that our findings might have been caused, at least in part, by transient changes in cerebral autoregulation. We need to conduct further studies to clarify whether current findings are induced by transient impairment of cerebral autoregulation.

This study has several limitations. The monitored systemic blood pressure and heart rate were not beat-to-beat measurements. Thus, we could not detect a transient drop in blood pressure in response to HOB manipulation. Lam et al. reported that the blood pressure may show a steep drop in response to gradual changes in head position (supine to 30°), although a blood pressure decline was observed in the head-up state [50]. Furthermore, the CBV paralleled blood pressure in the head-up position. These results supported our findings that neither blood pressure nor cerebral oxygenation parameters changed in response to HOB manipulation, at least according to the cohort-averaged assessment. Second, two consecutive NIRS measurements were needed for each participant because the TRS-10 system had only a single channel. Thus, we could not evaluate the cerebral oxygenation changes on the infarct and contra-infarct sides simultaneously. Furthermore, we could investigate forehead blood volume only within a narrow range of the prefrontal cortex. Multi-channel NIRS is currently in mainstream use and should be adopted in future studies to understand fluctuations in cerebral oxygenation in the entire brain in response to postural change. Third, we did not measure either endotidal carbon dioxide tension (EtCO_2_) or partial pressure of carbon dioxide (pCO_2_). It is known that CBF is influenced by CO_2_, and hypercapnia dilates cerebral arteries and arterioles and increased blood flow, whereas hypocapnia causes vasoconstriction and decreased blood flow [51,52]. Indeed, Kim, Y.S. et al. reported that orthostatic manipulation decreases EtCO_2_ from 40 mmHg to 35 mg in elder subjects [26]. Therefore, we cannot exclude that pCO_2_ could also affect NIRS metrics. Fourth, we did not assess cerebral autoregulation or differences in the CBV responses among NIRS and other modalities (e.g., TCD, PET). Thus, we could not offer reasons why a statistically significant correlation of changes in cerebral oxygenation parameters between each hemisphere in response to HOB manipulation was found at 7–10 days after AIS onset but not within 72 h.

## 5. Conclusions

HOB manipulation from 0° to 30° did not affect cohort-averaged hemodynamic parameters. The cohort-averaged cerebral oxygenation parameters also did not change in response to HOB elevation, although large interindividual cerebral oxygenation changes were seen. Although changes in cerebral oxygenation in response to HOB elevation had a laterality difference between the hemispheres within 72 h of AIS onset, the difference decreased, at least partially, 7–10 days after AIS onset, and this could have suggested a sign of cerebral blood flow recovery. These findings suggest that HOB 30° within 72 h might not always be a preferred head position in AIS patients. Further studies are needed to establish the safety and efficacy of NIRS-guided neurological rehabilitation in AIS in the future. 

## Figures and Tables

**Table 1:** Patients’ characteristics.

Characteristic	Value
Age, year	72.8 ± 11.3
Female sex, *n* (%)	13 (43.3)
Height (cm)	158.0 ± 11.6
Body weight (kg)	56.0 ± 14.4
Body mass index (kg/m^2^)	22.1 ± 3.1
Stroke side (right/left)	11/19
NIHSS score	7.6 ± 4.9
**TOAST classification, *n* (%)**	
Large-artery atherosclerosis	10 (33.3)
Small-vessel occlusion	10 (33.3)
Cardioembolism	6 (20.0)
Stroke of other determined etiology	4 (13.3)
Stroke of undetermined etiology	0 (0)
**Vascular territorial segmentation, *n* (%)**	
Anterior cerebral artery	6 (20.0)
Middle cerebral artery	17 (56.7)
Posterior cerebral artery	7 (20.3)
**Medical history *n* (%)**	
Hypertension	18 (60.0)
Diabetes mellitus	8 (26.7)
Dyslipidemia	22 (73.3)
Chronic atrial fibrillation	4 (13.3)
Tobacco use	12 (40.0)

Data are expressed as means ± Standard deviation unless otherwise stated; TOAST—Trial of Org 10172 in Acute Stroke Treatment; NIHSS—National Institutes of Health Stroke Scale.

**Table 2:** Comparisons of the systemic blood pressure and heart rate in response to HOB manipulation.

Parameters	Item Measured	HOB 0°	HOB 30°	Difference	*p*
**First Measurement**					
**Contra-infarct Side**	sBP (mmHg)	137.6 ± 20.9	137.7 ± 19.8	0.1 ± 4.1	0.90
	dBP(mmHg)	75.9 ± 11.7	76.9 ± 10.6	1.0 ± 5.4	0.91
	HR (bpm)	73.5 ± 10.4	73.5 ± 9.7	0.0 ± 3.5	0.95
**Infarct Side**	sBP (mmHg)	137.9 ± 20.6	137.3 ± 19.8	−0.6 ± 3.7	0.39
	dBP (mmHg)	75.9 ± 11.2	76.4 ± 10.9	0.5 ± 4.6	0.63
	HR (bpm)	73.6 ± 11.0	73.4 ± 11.4	−0.2 ± 3.6	0.86
**Second Measurement**					
**Contra-Infarct Side**	sBP (mmHg)	128.9 ± 16.5	129.8 ±15.0	0.9 ± 5.1	0.53
	dBP (mmHg)	71.0 ± 9.0	71.1 ± 8.6	0.0 ± 3.9	0.51
	HR (bpm)	75.6 ± 8.1	76.5 ± 7.8	0.9 ± 3.9	0.36
**Infarct Side**	sBP (mmHg)	129.5 ± 15.1	128.7 ± 15.6	−0.8 ±5.3	0.10
	dBP (mmHg)	71.0 ± 8.4	70.3 ± 8.3	−0.7 ± 4.6	0.24
	HR (bpm)	74.8 ± 8.4	75.1 ± 7.7	0.3 ± 3.1	0.21

Data are expressed as means ± Standard deviation; HOB—head-of-bed, sBP—systolic blood pressure; dBP—diastolic blood pressure; HR—heart rate; bpm—beats per minute; mmHg—millimeters of mercury.
